# Comparison of Antibacterial and Immunological Properties of Mesenchymal Stem/Stromal Cells from Equine Bone Marrow, Endometrium, and Adipose Tissue

**DOI:** 10.1089/scd.2017.0241

**Published:** 2018-10-31

**Authors:** Yennifer Cortés-Araya, Karin Amilon, Burgunde Elisabeth Rink, Georgina Black, Zofia Lisowski, Francesc Xavier Donadeu, Cristina L. Esteves

**Affiliations:** ^1^ The Roslin Institute and R(D)SVS, University of Edinburgh, Edinburgh, United Kingdom.; ^2^ The Euan Macdonald Centre for Motor Neurone Disease Research, University of Edinburgh, Edinburgh, United Kingdom.

**Keywords:** mesenchymal stem/stromal cells, veterinary, horse, adipose tissue, endometrium, bacteria

## Abstract

Equine mesenchymal stem/stromal cells (MSCs) are multipotent cells that are widely used for treatment of musculoskeletal injuries, and there is significant interest in expanding their application to nonorthopedic conditions. MSCs possess antibacterial and immunomodulatory properties that may be relevant for combating infection; however, comparative studies using MSCs from different origins have not been carried out in the horse, and this was the focus of this study. Our results showed that MSC-conditioned media attenuated the growth of *Escherichia coli*, and that this effect was, on average, more pronounced for endometrium (EM)-derived and adipose tissue (AT)-derived MSCs than for bone marrow (BM)-derived MSCs. In addition, the antimicrobial lipocalin-2 was expressed at mean higher levels in EM-MSCs than in AT-MSCs and BM-MSCs, and the bacterial component lipopolysaccharide (LPS) stimulated its production by all three MSC types. We also showed that MSCs express interleukin-6 (IL-6), IL-8, monocyte chemoattractant protein-1, chemokine ligand-5, and Toll-like receptor 4, and that, in general, these cytokines were induced in all cell types by LPS. Low expression levels of the macrophage marker colony-stimulating factor 1 receptor were detected in BM-MSCs and EM-MSCs but not in AT-MSCs. Altogether, these findings suggest that equine MSCs from EM, AT, and BM have both direct and indirect antimicrobial properties that may vary between MSCs from different origins and could be exploited toward improvement of regenerative therapies for horses.

## Introduction

Equine mesenchymal stem/stromal cells (MSCs), obtained typically from bone marrow (BM) and adipose tissue (AT), have been used clinically for more than a decade. Although great progress has been made toward their characterization [1–9], there is still a lack of understanding regarding their antibacterial and immune-related properties. Antibiotic resistance is emerging as a major health risk for both humans and animals, and new strategies to ameliorate this problem are in great need.

The regenerative potential of MSCs derives not only from their ability to differentiate into mature mesenchymal cell lineages, but also from a variety of immunomodulatory effects exerted by these cells [10–14] that, importantly, contribute to combating infection and determine clinical outcome in patients receiving MSCs. Moreover, human and mouse MSCs have been shown to express several antimicrobials and to be able to attenuate bacterial growth [12,15–17], a finding that has been recently extended to MSCs derived from equine blood [18].

In humans, MSCs can have different inflammatory phenotypes depending on the extracellular milieu [19]. Interestingly, in a low inflammatory environment or upon activation of Toll-like receptor 4 (TLR4) by bacterial lipopolysaccharide (LPS), MSCs polarize to a proinflammatory state (MSC1), characterized by increased production of chemokines and cytokines that may recruit and activate immune effector cells [19]. In a different environment, MSCs may display an anti-inflammatory phenotype (MSC2).

Limited numbers of studies have investigated the immunological properties of equine MSCs [4,20,21,41]. Moreover, there is accumulating evidence that MSCs from different tissue sources differ in their TLR-expression profiles and response to inflammatory stimuli [22]. In this study, we investigated the antibacterial and immunomodulatory properties of MSCs from three different equine tissues sources, namely, BM and AT, the two most common sources of clinical MSCs, and endometrium (EM), a promising novel source of therapeutic MSCs [23,24].

## Materials and Methods

### Sourcing of MSCs

BM-MSCs, AT-MSCs, and EM-MSCs were obtained each from three horses as described [5,23], and were characterized following the criteria established by the International Society for Cellular Therapy for MSCs [25]. All animal procedures were carried out according to the UK Home Office Animals (Scientific Procedures) Act 1986 with approval by the Ethical Review Committee, University of Edinburgh.

BM-MSCs were obtained by aspiration of the sternum marrow, centrifugation on a density gradient, and culture of the resulting mononucleated cell layer.

AT-MSCs were obtained by mincing subcutaneous AT followed by collagenase II (1 mg/mL; Gibco-Thermo Fisher Scientific, Paisley, UK)/bovine serum albumin (BSA; 3.5%) digestion at 37°C under agitation (100 rpm). Digestion was stopped by addition of Dulbecco's modified Eagle's medium (DMEM) 20% fetal bovine serum (FBS; Gibco-Thermo Fisher Scientific), the fat layer removed and cells were further washed and seeded.

To harvest EM-MSCs [23], the tissue was washed and minced and then dissociated in DMEM/F-12 containing 0.1% BSA, 0.5% collagenase I, 40 μg/mL deoxyribonuclease type I (Sigma-Aldrich, Irvine, UK), and 1% penicillin/streptomycin (P/S) for 40 min at 37°C. Stromal cells were separated by negative selection of epithelial cells using Muc1-beads, filtered, washed, and cultured. MSCs were expanded in DMEM 10% FBS and 1% P/S at 37°C in a humidified atmosphere containing 5% carbon dioxide (CO_2_). Alveolar macrophages were obtained by bronchoalveolar lavage from adult horses and cryopreserved as previously described [26]. Before use, cells were thawed and seeded in 24-well plastic plates (Nunc, Thermo Scientific) at 1 × 10^6^ cells/mL in complete medium (RPMI-1640 with GlutaMAX^™^-I Supplement; Invitrogen), 1% P/S (Invitrogen), and 20% heat-inactivated horse serum (Sigma-Aldrich) and incubated overnight at 37°C and 5% CO_2_. The next day, nonadherent cells were removed and the medium was replaced.

### Bacterial growth

Conditioned medium (CM; DMEM 10% FBS) was harvested from MSCs (BM, EM, and AT; 70,000 cells/well in 12-well plates) after culture for 16 h at 37°C. CM was spun to remove cell debris and kept at −80°C. *Escherichia coli* ZAP198 was inoculated in BM-CM, EM-CM, and AT-CM for 16 h at 37°C, and colony-forming units (cfu/mL) were obtained by serial dilutions in Luria–Bertani (LB) agar plates. Bacteria grown in DMEM 10% FBS and LB served as positive controls.

### Gene expression analyses

Total RNA was extracted using Trizol's protocol and was reverse transcribed using Superscript III (18080-044; Invitrogen-Thermo Fisher Scientific). Transcript levels were quantified using an MX3005P quantitative polymerase chain reaction (qPCR) system (Stratagene) with primers listed in Table 1 and SensiFAST SYBR Lo-ROX kit (Bioline). Data were analyzed using Stratagene MxPro software and the messenger RNA (mRNA) levels were determined relative to a standard curve (generated from sample pools) that was run simultaneously.

**Table 1.** Gene and Respective Pair of Primers Used for Quantitative Polymerase Chain Reaction

*Gene*	*Sense primer sequence (5′-3′)*	*Antisense primer sequence (5′-3′)*	*References*
*IL-6*	GGACCACTACTCACCACTGC	CCCAGATTGGAAGCATCCGT	
*MCP-1*	ATATCAGGGGGCATTTAGGG	ATTGGCCAAGGAGATCTGTG	
*CCL5*	CAGTCGTCTTTGTCACCCGA	GGTTCGAGATGCCCTCCAAT	
*LCN2*	CCACAGCTACAACGTCACCT	GGCTGGGAACTTGGGATGAA	
*IL-8*	CTTTCTGCAGCTCTGTGTGAAG	GCAGACCTCAGCTCCGTTGAC	[39]
*TLR4*	GCCACCTGTCAGATTAGCAAGA	AGAACTGCTATGACAGAAACCATGA	[28]
*CSF1-R*	GAAATACGTCCGCAGGGACA	GACACGGGTCTCATCTCCAC	
*TNF-α*	CCTGTAGCCCATGTTGTAGCA	GGACCTGGGAGTAGATGAGGT	
*18S*	GCTGGCACCAGACTTG	GGGGAATCAGGGTTCG	
*GAPDH*	CAGAACATCATCCCTGCTTC	ATGCCTGCTTCACCACAATTC	

CCL5, chemokine ligand-5; CSF1-R, colony-stimulating factor 1 receptor; GAPDH, glyceraldehyde 3-phosphate dehydrogenase; IL-6, interleukin-6; IL-8, interleukin-8; LCN2, lipocalin-2; MCP-1, monocyte chemoattractant protein-1; TLR4, Toll-like receptor 4; TNF-α, tumor necrosis factor α.

Results were normalized to 18S ribosomal RNA (rRNA) and glyceraldehyde 3-phosphate dehydrogenase (GAPDH), and expressed as arbitrary units (AU). Reverse transcriptase-negative and no template control samples were included in each run as negative controls. For the measurement of colony-stimulating factor 1 receptor (CSF1-R) mRNA levels, complementary DNA from equine macrophages and keratinocytes [27] were included as positive and negative controls, respectively.

### LPS stimulation experiments

MSCs at passages 4–6 were seeded at a density of 70,000 cells per well in 12-well plates (Nunc, Thermo Scientific). After 24 h, the cell culture medium was removed and replaced with the medium containing 0 or 0.1 μg/mL LPS (Sigma-Aldrich). After 16 h of incubation, the wells were washed with phosphate-buffered saline (PBS) and cells harvested into Trizol (Thermo Fisher Scientific), frozen and stored at −80°C before further analysis. Cell stimulation conditions, 0.1 μg/mL LPS for 16 h, were chosen based on results from previous time-course and LPS dose–response trials (Supplementary Fig. S1; Supplementary Data are available online at www.liebertpub.com/scd).

### Immunocytochemistry

Cultured cells were washed with PBS and fixed with paraformaldehyde for 15 min, and kept at 4°C until use. Cells were permeabilized using methanol:acetone (1:1) followed by incubation with protein blocking solution (Insight Biotechnology). Cells were then incubated with primary antibodies against lipocalin-2 (ab41105; Abcam, Cambridge, UK) or TLR4 (sc-12511; Santa Cruz Biotechnology, Paso Robles, CA) for 16 h at 4°C, and then with secondary antibodies [anti-rabbit and anti-goat immunoglobulin G (10042 and 11057; both from Invitrogen-Thermo Fisher Scientific) conjugated to the AF568 fluorochrome]. The primary antibodies have been previously used to detect lipocalin-2 and TLR4 in equine samples [18,26]. Samples were then mounted in fluoroshield with 4′,6-diamidino-2-phenylindole (DAPI; F6057; Sigma, St Louis, MO) and micrographs taken with a camera connected to a Zeiss Axiovert 25 microscope. The same settings were used for all pictures taken for each antibody. Cells incubated with secondary antibody only were used as negative controls.

### Enzyme-linked immunosorbent assay

Monocyte chemoattractant protein-1 (MCP-1) concentrations in cell culture supernatants were analyzed by enzyme-linked immunosorbent assay (ELISA; ELE-MCP-1; Cambridge Bioscience, Cambridge, UK) according to the manufacturer's protocol. In brief, samples and standards were added to a 96-well plate coated with anti-equine MCP-1 antibody, and incubated for 2.5 h at room temperature with gentle agitation. Samples were washed and incubated with biotinylated antibody for 1 h. After addition of horseradish peroxidase-conjugated streptavidin and 3,3,5,5′-tetramethylbenzidine subtract reagent, signal detection was performed at 450 nm. Equine MCP-1 protein provided in the kit was used to produce the standard curve.

### Statistical analysis

The effects of LPS on MSCs from different tissues were analyzed by two-way analysis of variance including “tissue,” “treatment,” “tissue × treatment” interaction and “animal” nested within “tissue” using the Minitab 17 statistical software (Minitab, Inc.). Fisher's exact test was used for post hoc comparisons. Data were log-transformed before analyses to comply with normality criteria. Significance was set at *P* < 0.05.

## Results

### Equine MSCs attenuate bacterial growth and express lipocalin-2

**FIG. 1.**
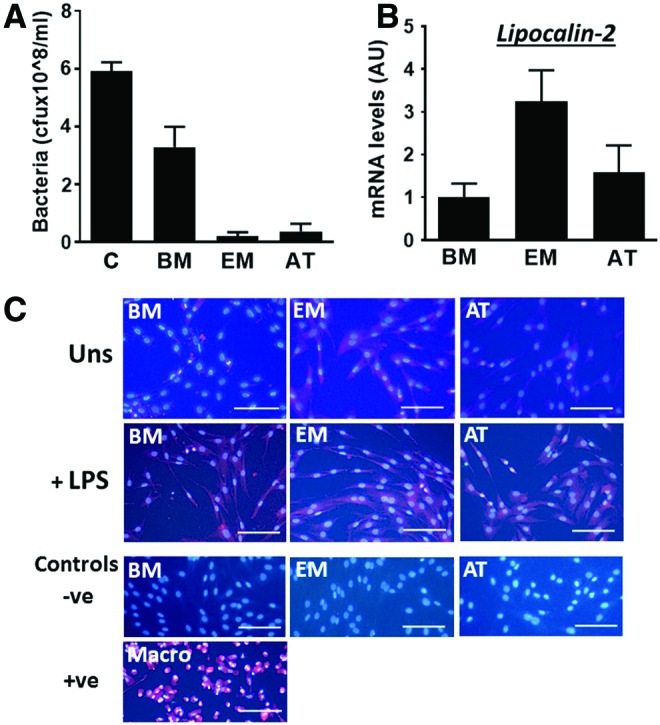
**(A)** *Escherichia coli* growth, indicated as cfu/mL, 16 h after bacteria inoculation in CMs from BM-MSCs, EM-MSCs, or AT-MSCs or in growth medium only (positive control, C). **(B)** Lipocalin-2 transcript levels in equine MSCs from BM, EM, or AT. Data are given as mean ± SEM (*n* = 3 horses/tissue type). Mean mRNA levels in BM-MSC samples were set to 1. **(C)** Fluorescence ICC of unstimulated (Uns) or LPS-stimulated MSCs from BM, EM, or AT, with lipocalin-2 antibody. Negative controls (−ve) were produced with LPS-stimulated cells incubated with secondary antibody only, and positive control (+ve) resulted from staining of alveolar macrophages with lipocalin-2 antibody. Lipocalin-2 is indicated by *red* signal and DAPI-stained nuclei are shown in *blue*. Pictures were taken in an Axiovert 25 inverted microscope. Scale bars, 100 μm. AT, adipose tissue; AU, arbitrary units; BM, bone marrow; cfu, colony-forming units; DAPI, 4′,6-diamidino-2-phenylindole; EM, endometrium; ICC, immunocytochemistry; LPS, lipopolysaccharide; mRNA, messenger RNA; MSCs, mesenchymal stem/stromal cells; SEM, standard error of the mean. Color images available online at www.liebertpub.com/scd

To assess direct effects of MSCs on bacterial growth, *E. coli* were inoculated in CMs from BM-MSCs, EM-MSCs, and AT-MSCs. All three CMs attenuated bacterial growth, although, on average, EM-MSC and AT-MSC had more pronounced effects than BM-MSC media (Fig. 1A). We then determined whether equine MSCs expressed antimicrobial genes. All cell types expressed lipocalin-2, both at the mRNA (Fig. 1B) and protein (Fig. 1C) levels, but not other antimicrobial genes assayed, namely, LL-37 and β-defensin 1. Interestingly, EM-MSCs expressed lipocalin-2 at higher mean levels (≥2-fold) than BM-MSCs and AT-MSCs (Fig. 1B). Moreover, fluorescence immunocytochemistry (ICC) showed increased lipocalin-2 protein signal in MSCs stimulated with LPS (Fig. 1C).

### Equine MSCs express immunomodulatory genes

**FIG. 2.**
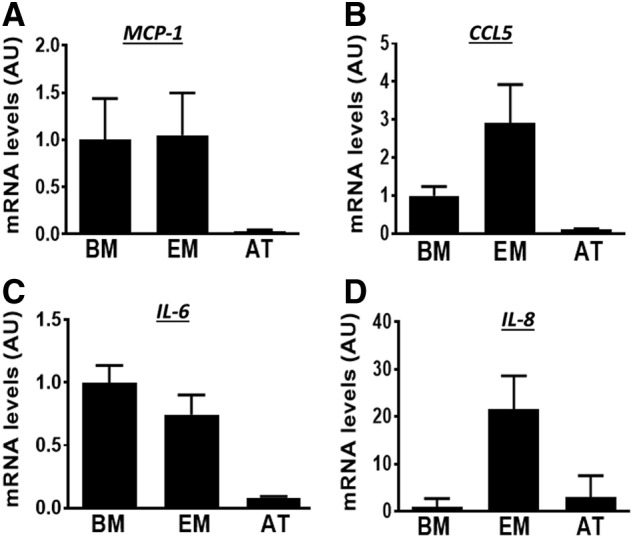
Transcript levels of **(A)** MCP-1, **(B)** CCL5, **(C)** IL-6, and **(D)** IL-8 in MSCs from BM, EM, and AT. Data are given as mean ± SEM (*n* = 3 horses/tissue type). Mean mRNA levels of BM-MSC samples was set to 1. CCL5, chemokine ligand-5; IL-6, interleukin-6; IL-8, interleukin-8; MCP-1, monocyte chemoattractant protein-1.

To examine the immunomodulatory properties of the three types of MSCs, we determined the expression of genes including the cytokines, MCP-1, chemokine ligand-5 (CCL5), interleukin-6 (IL-6), and IL-8. Samples had detectable levels of all cytokines analyzed (Fig. 2). Mean MCP-1 mRNA levels were higher (≥2.9-fold) in BM-MSCs and EM-MSCs than in AT-MSCs (Fig. 2A). Similarly, CCL5 and IL-6 were expressed at relatively much lower levels in AT-MSCs (Fig. 2B, C), whereas mean IL-8 expression was much higher (≥7-fold) in EM-MSCs than in either BM-MSCs or AT-MSCs (Fig. 2D). Thus, AT-MSCs expressed the lowest levels of all immunomodulatory genes analyzed.

### MSCs are responsive to LPS

**FIG. 3.**
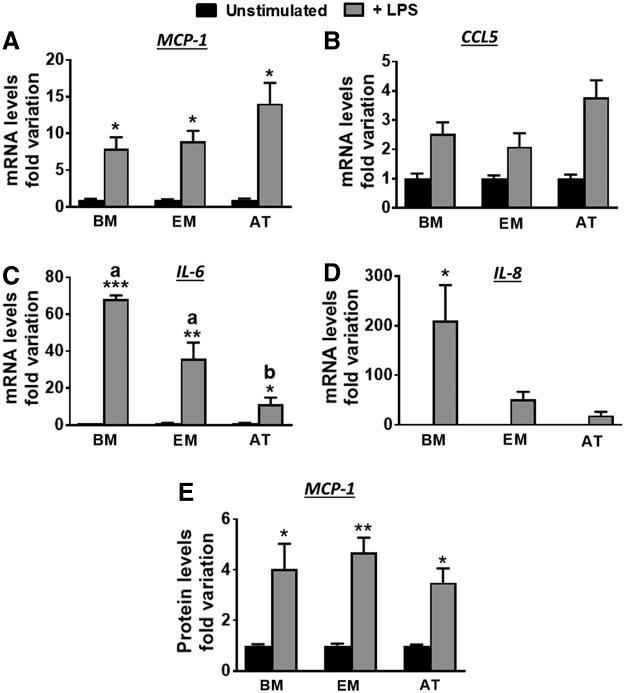
Fold change in MCP-1 **(A)**, CCL5 **(B)**, IL-6 **(C)**, and IL-8 **(D)** mRNA levels, and MCP-1 protein levels **(E)** after LPS stimulation (a *gray bars*) of MSCs from BM, EM, and AT. In each case, data (given as mean ± SEM; *n* = 3 horses/tissue type) were normalized to mean mRNA levels in unstimulated cells (*black bars*). *P < 0.05, **P < 0.001, and ***P < 0.0001 indicate differences between unstimulated and stimulated cells, whereas different *superscripts* (a, b) show significant differences between cell types (*P* < 0.03).

We then examined the effects of stimulation with LPS on the expression of immunomodulatory genes by MSCs. LPS induced a dramatic increase (≥7-fold; *P* < 0.05) in MCP-1 mRNA levels across all three cell types (Fig. 3A). An increase in CCL5 mRNA was also observed, although this was not significant for any cell type (Fig. 3B). In contrast, for IL-6, a graded response to LPS was observed across cells with lower fold induction in AT-MSCs (*P* < 0.05) than in BM-MSCs (*P* < 0.0001) or EM-MSCs (*P* < 0.001) (Fig. 3C, D). A similar graded response in IL-8 mRNA was observed with the LPS induction being significant (*P* < 0.05) only in BM-MSCs (*P* < 0.05).

To confirm the results from qPCR, we analyzed MCP-1 levels in CMs using a commercial available ELISA kit that recognizes the equine protein. In agreement with mRNA data (Fig. 3A), MCP-1 protein was significantly induced in response to LPS (Fig. 3E) in BM-MSCs (0.4 ± 0.07 vs. 1.4 ± 0.3 ng/mL for unstimulated and LPS stimulated, respectively, *P* < 0.05), EM (0.2 ± 0.03 vs. 0.9 ± 0.07, *P* < 0.001), and AT (0.09 ± 0.02 vs. 0.3 ± 0.02, *P* < 0.05).

**FIG. 4.**
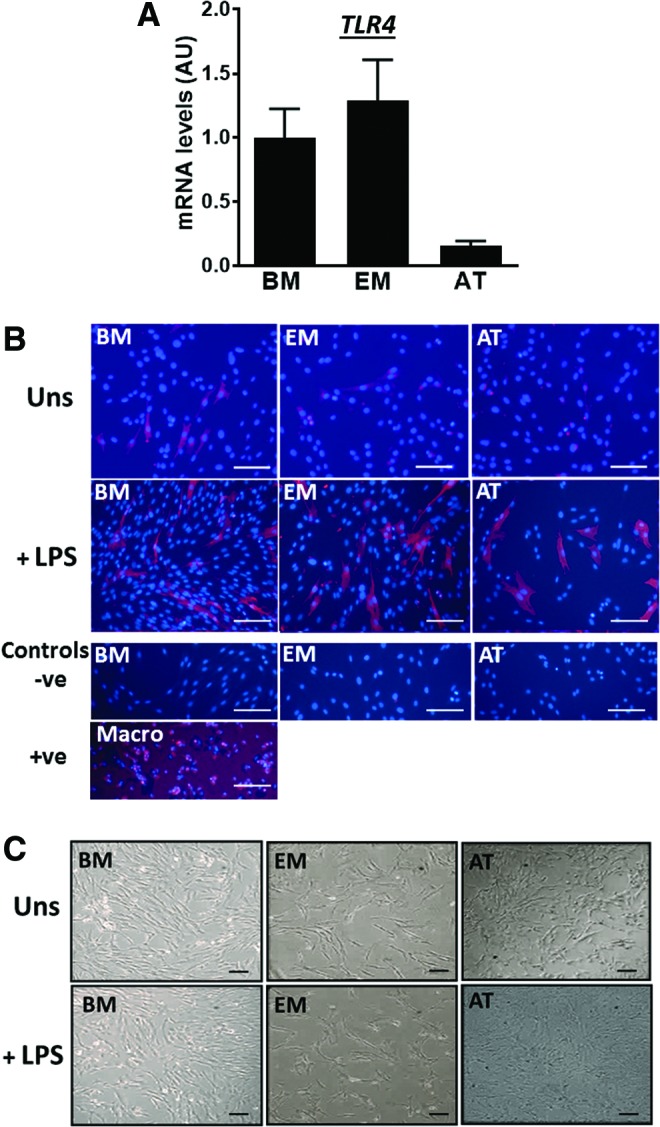
**(A)** TLR4 expression measured by qPCR in unstimulated MSCs from BM, EM, and AT. Data are given as mean ± SEM (*n* = 3 horses/tissue type). Mean mRNA levels in BM-MSC samples were set to 1. **(B, C)** Photomicrographs showing **(B)** fluorescence immunostaining of TLR4 (in *red*) and **(C)** bright field images of MSCs from BM, EM, and AT before (unstimulated, Uns) and after a 16 h simulation with LPS (0.1 μg/mL). Negative controls (−ve) correspond to LPS-stimulated cells incubated with secondary antibody only, and positive control (+ve) to alveolar macrophages incubated with TLR4 antibody. All pictures were taken in an Axiovert 25 inverted microscope. Scale bars, 100 μm. qPCR, quantitative polymerase chain reaction; TLR4, Toll-like receptor 4. Color images available online at www.liebertpub.com/scd

We also quantified the relative expression of TLR4 [28], a cognate LPS receptor, in MSC preparations both at the mRNA (Fig. 4A) and protein (Fig. 4B) levels. TLR4 was detected in unstimulated cells, although at variable levels; mean mRNA levels were higher (≥6.5-fold) in BM-MSCs and EM-MSCs than those in AT-MSCs (Fig. 4A), consistent with protein data (upper row in Fig. 4B). Cells were then stimulated with LPS for 16 h. This did not produce any apparent changes in cell morphology or cell numbers (Fig. 4C), but results from fluorescence ICC indicated increased levels of TLR4 protein in response to LPS in all MSC types (Fig. 4B).

### Low levels of CSF1-R are present in BM-MSC and EM-MSC preparations

**FIG. 5.**
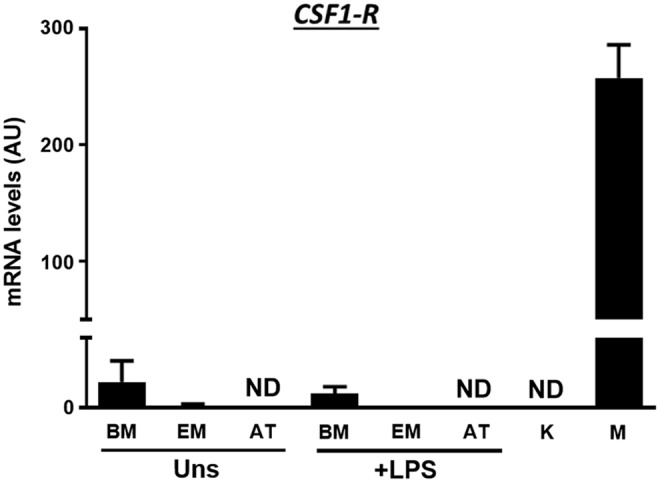
mRNA levels of the macrophage marker, CSF1-R, in unstimulated (Uns) and LPS-stimulated (0.1 μg/mL LPS for 16 h) MSCs from equine BM, EM, and AT. Keratinocyte (K) and macrophage (M) samples were used as negative and positive controls, respectively. Results are given as mean + SEM. *N* = 3 horses per tissue type. CSF1-R, colony-stimulating factor 1 receptor; ND, not detected.

To assess whether contamination of MSCs with immune cells such as macrophages, as reported in other studies [29], may have influenced our results, we measured the expression of the macrophage-specific gene, CSF1-R, in MSC preparations and compared these with the levels expressed by macrophages (positive control) and keratinocytes (negative control). CSF1-R was detected at very low levels in BM-MSCs and EM-MSCs (<700-fold lower than in macrophage samples) but not in AT-MSCs or keratinocytes. Also, LPS stimulation did not induce changes in CSF1-R expression, although this finding did not completely rule out the presence of macrophages in MSC preparations (Fig. 5).

## Discussion

Antimicrobial resistance poses a growing threat for both animal and human health, requiring the identification of novel approaches to fight microbial infection. Studies over the past 10 years have demonstrated the immunomodulatory nature of MSCs including direct antimicrobial effects [12,13,15,17,18,30–32], providing an attractive therapeutic tool alternative or complementary to the use of antibiotics. The identification of novel MSC sources has also represented a step forward in that regard. For example, although BM and AT are well-established sources of clinical MSCs in both humans and animals, EM is now emerging as a promising alternative source with defined cell differentiation and immunomodulatory properties [23,24,30,33]. It is thus critical to compare the immunomodulatory properties of MSCs across different tissues to identify the most optimal source(s) for particular clinical application. In that regard, this is to our knowledge the first study to simultaneously compare the properties of MSCs from BM, EM, and AT.

Several studies in humans and rodents [16,34] have shown that MSCs are able to attenuate microbial growth and that this effect can be attributed to the production of antimicrobials. These findings were recently extended for the first time to the horse, specifically to equine blood-derived MSCs [18]. In this study, we report that several common sources of clinical MSCs in the horse are able to inhibit bacterial growth and that this effect varies between cell sources, being apparently higher for EM-MSCs and AT-MSCs than for BM-MSCs. Moreover, we show that all three cell sources express lipocalin-2, and that EM-MSCs express, on average, the highest levels.

In agreement with our results, a recent study showed that production of lipocalin-2, among other antimicrobial peptides, contributed to the antibacterial effects of equine blood-derived MSCs that did not produce β-defensin [18]. In contrast to our results, however, blood-derived MSCs did produce substantial LL-37, a finding that may reflect tissue-specific differences in antimicrobial production by MSCs. In that regard, human BM-MSCs and umbilical cord blood (UCB)-MSCs have been shown to reduce bacterial growth through the secretion of LL-37 and β-defensin 2, respectively [15,16,34]. Our observation of apparent highest lipocalin-2 expression in EM-MSCs may be linked to the fact that, unlike BM or AT, EM provides a natural body barrier against infection. EM-MSCs could thus provide distinct benefits for clinical use.

In addition to lipocalin-2, equine MSCs from all sources examined expressed the immunomodulatory genes, MCP-1, IL-6, IL-8, and CCL5, suggesting that these cytokines may contribute to the reported ability of equine MSCs to limit infection indirectly by recruiting and activating immune cells [32,35]. Of these four cytokines, MCP-1 and CCL5 have to our knowledge not been reported previously to be expressed in equine MSCs. Of interest, expression of all the above immunomodulatory genes was generally reduced in AT-MSCs relative to BM-MSCs and EM-MSCs.

Although we do not have an explanation for this, expression of CSF1-R was lowest in AT-MSCs, suggesting that contamination by macrophages, even at low levels, may have possibly contributed to the elevated cytokine expression in BM and EM. Indeed, the presence of contaminating leukocyte populations in MSC preparations probably contributes to the variability in clinical outcomes reported with the use of these cells. On the contrary, it has also been reported that BM mesenchymal progenitor cells can originate from CD14^+^ cells [36]. Overall, this highlights the need for more robust characterization of MSC populations.

In the context of infection and tissue repair, the inflammatory microenvironment and specific pattern of TLR expression in effector cells determine both the interactions between MSCs and immune cells and the outcome of tissue regeneration approaches using MSCs [37]. In agreement with the findings with human BM-MSCs and AT-MSCs [38], and equine UCB-MSCs [20], we detected expression of TLR4 in MSCs from all sources, consistent with their responsiveness to LPS. Polarization into a proinflammatory MSC1 phenotype in response to TLR4 activation is marked by increased secretion of immune effector-recruiting cytokines and chemokines [19]. In this study, in general, the expression of MCP-1, IL-6, IL-8, and CCL5 increased in response to LPS stimulation, consistent with reports with human AT-MSCs and BM-MSCs [39,40] and, for those cytokines that have been examined (IL-6 and IL-8) in equine BM-MSCs [41].

In summary, our results suggest that MSCs from different sources have both antimicrobial activity and constitutively produce lipocalin-2 that may physiologically contribute to innate immune responses, particularly in the case of EM-MSCs. However, the largest component of the reported in vivo antibacterial activity of MSCs probably involves indirect activation of immune effector cells. This conclusion is in line with observations that LPS-stimulated human MSCs induce the expression of IL-6 and IL-8 and enhance the activation and phagocytic activity of polymorphonuclear neutrophils [42]. Overall our findings suggest that equine MSCs, particularly EM-MSCs, could be of benefit for reducing and limiting infection. However, further studies will be necessary to assess the antibacterial activity of these cells in an in vivo context so that new strategies can be developed to diversify their use and increase their therapeutic efficiency.

## Supplementary Material

Supplemental data
