# Supplementary material for: Comparison of Antibacterial and Immunological Properties of Mesenchymal Stem/Stromal Cells from Equine Bone Marrow, Endometrium, and Adipose Tissue
Source: Stem Cells Dev. 2018 Oct 31;27(21):1518–25. doi: 10.1089/scd.2017.0241 (PMC6209426; doi:10.1089/scd.2017.0241)
Supplement: Supplemental data [file Supp_Fig1.pdf]

Supplementary Data

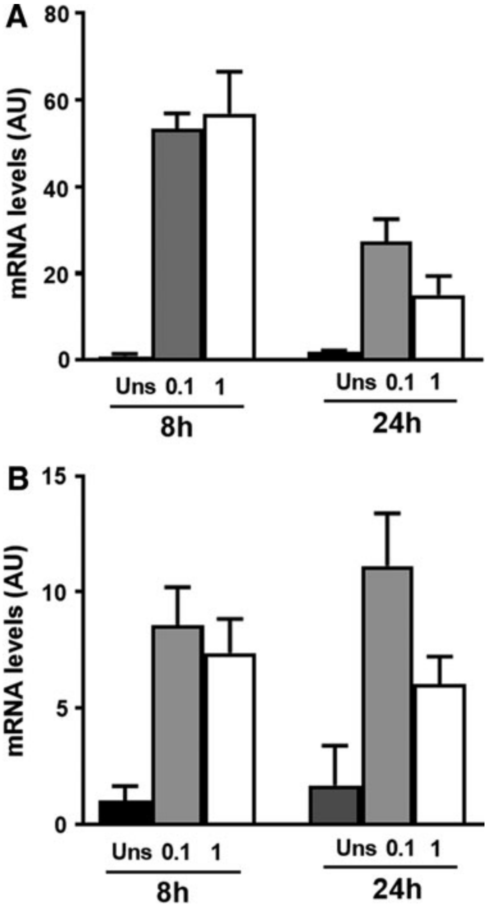

SUPPLEMENTARY FIG. S1. IL-6 mRNA levels from BM-MSCs **(A)** and AT-MSCs **(B)** that were unstimulated (Uns) or stimulated with 0.1 or 1 μg/mL LPS for 8 or 24 h. Results (mean ± SEM) are from one cell preparation of each type assayed in triplicate. AT, adipose tissue; AU, arbitrary units; BM, bone marrow; IL-6, interleukin-6; LPS, lipopolysaccharide; mRNA, messenger RNA; MSCs, mesenchymal stem/stromal cells; SEM, standard error of the mean.
